# A systematic concept analysis of ‘technology dependent’: challenging the terminology

**DOI:** 10.1007/s00431-020-03737-x

**Published:** 2020-07-24

**Authors:** Maria Brenner, Denise Alexander, Mary Brigid Quirke, Jessica Eustace-Cook, Piet Leroy, Jay Berry, Martina Healy, Carmel Doyle, Kate Masterson

**Affiliations:** 1 School of Nursing and Midwifery, Trinity College Dublin, The University of Dublin, 24 D’Olier Street, Dublin 2, Ireland; 2 Trinity College Dublin, The University of Dublin, Dublin, Ireland; 3 Pediatric Intensive Care Unit & Pediatric Procedural Sedation Unit, Maastricht UMC and Faculty of Health, Life Sciences & Medicine, Maastricht University, Maastricht, Netherlands; 4 Department of Medicine and Division of General Pediatrics, Boston Children’s Hospital and Harvard Medical School, Boston, MA USA; 5 Department of Paediatric Anaesthesia, Paediatric Critical Care Medicine and Paediatric Pain Medicine, Children’s Health Ireland Crumlin, Dublin, Ireland; 6 School of Medicine, Faculty of Health Sciences, Trinity College Dublin, the University of Dublin, Dublin, Ireland; 7 Paediatric Intensive Care Unit, The Royal Children’s Hospital, Melbourne, Australia

**Keywords:** Child, Complex needs, Concept analysis, Family, Technology dependence

## Abstract

There are an increasing number of children who are dependent on medical technology to sustain their lives. Although significant research on this issue is taking place, the terminology used is variable and the concept of technology dependence is ill-defined. A systematic concept analysis was conducted examining the attributes, antecedents, and consequences of the concept of technology dependent, as portrayed in the literature. We found that this concept refers to a wide range of clinical technology to support biological functioning across a dependency continuum, for a range of clinical conditions. It is commonly initiated within a complex biopsychosocial context and has wide ranging sequelae for the child and family, and health and social care delivery.

*Conclusion*: The term technology dependent is increasingly redundant. It objectifies a heterogenous group of children who are assisted by a myriad of technology and who adapt to, and function with, this assistance in numerous ways.

**Table:** 

**What is Known:** • *There are an increasing number of children who require medical technology to sustain their life, commonly referred to as technology dependent. This concept analysis critically analyses the relevance of the term technology dependent which is in use for over 30 years.*	
**What is New:** • *Technology dependency refers to a wide range of clinical technology to support biological functioning across a dependency continuum, for a range of clinical conditions. It is commonly initiated within a complex biopsychosocial context and has wide-ranging sequelae for the child and family, and health and social care delivery.* • *The paper shows that the term technology dependent is generally portrayed in the literature in a problem-focused manner.* • *This term is increasingly redundant and does not serve the heterogenous group of children who are assisted by a myriad of technology and who adapt to, and function with, this assistance in numerous ways. More appropriate child-centred terminology will be determined within the TechChild project.*

**What is Known:**

• *There are an increasing number of children who require medical technology to sustain their life, commonly referred to as technology dependent. This concept analysis critically analyses the relevance of the term technology dependent which is in use for over 30 years.*

**What is New:**

• *Technology dependency refers to a wide range of clinical technology to support biological functioning across a dependency continuum, for a range of clinical conditions. It is commonly initiated within a complex biopsychosocial context and has wide-ranging sequelae for the child and family, and health and social care delivery.*

• *The paper shows that the term technology dependent is generally portrayed in the literature in a problem-focused manner.*

• *This term is increasingly redundant and does not serve the heterogenous group of children who are assisted by a myriad of technology and who adapt to, and function with, this assistance in numerous ways. More appropriate child-centred terminology will be determined within the TechChild project.*

## Introduction

There are an increasing number of children who require medical technology to sustain their life [1–4] and, in response, an expanding array of medical technology available. This paper analyses the concept of ‘technology dependent’, the term commonly used to refer to these children. The current prevalence and rate of increase in the number of children who are technology dependent is difficult to determine as they depend on a number of interrelated factors including: the prevalence of particular conditions; medical and surgical interventions chosen; and finance and policies for care delivery across and within countries [5–7]. The term technology dependent stems primarily from the phrase ‘technology dependence’ which was coined 30 years ago by the Office of Technology Assessment (US) [8], describing ‘a medical device to compensate for the loss of a vital body function and substantial ongoing nursing care to avert death or further disability’*.* To the best of our knowledge, this is the first systematic concept analysis to examine the contemporary relevance and utility of this terminology as it is currently portrayed in the literature. This is important for two key reasons: the array of medical technology now available means that the broad term of ‘technology dependent’ has the potential to conjure up a multitude of clinical scenarios; and the objectifying nomenclature does not reflect a child-centric approach. This paper is part of a larger body of research, the TechChild project, funded by the European Research Council. The purpose of this research is to explore influences on the initiation of technology dependence required to sustain a child’s life and to identify more appropriate child-centred terminology in an evidenced-based manner.

An evolutionary concept analysis was employed as it seeks to examine the cluster of key characteristics that through common use, collectively form the *real* definition of a concept [9]. According to Rodgers [9], it is necessary to understand the antecedents (phenomena usually found prior to concept occurrence), the attributes of the concept, and the consequences that follow as a result. Without a clear conceptual foundation, there is an ambiguity which in turn can compromise the quality of research or theory construction as the area develops [10].

## Methods

Rodgers’ evolutionary method [9] was used to systematically analyse the concept of technology dependence in the scientific literature. This method is particularly well suited to this issue given the changing and dynamic nature of advances in medical technology. Alternative approaches to concept analysis are founded in a realist paradigm, in which a reductionist approach focuses on defining a concept as a static entity. Rodgers’ relativist stance, in comparison, seeks to identify how a concept is portrayed in the literature using an inductive approach, while acknowledging that any understanding of a concept is evolutionary as it is influenced by dynamic contextual factors, which may be disciplinary, cultural, or theoretical [9]. Rodgers’ approach to concept analysis includes a set of core activities, which can be carried out simultaneously and not necessarily in a linear manner (Table 1). Analysis seeks to identify what is common, the purpose of which is to identify data that is relevant to the attributes of the concept and its contextual features. Thematic analysis identifies major themes presented in the literature.

**Table 1:** Rodgers’ approach to concept analysis

Activities	
1.	Identify the concept of interest and associated expressions (including surrogate terms)
2.	Identify and select an *appropriate realm* for data collection
3.	*Collect* relevant data
4.	*Analyse* the data
5.	Identify an *exemplar* of the concept, if appropriate
6.	Identify *implications*, *hypotheses*, and *implications* for further development of the concept

### Data sources and search strategy

**Fig. 1:**
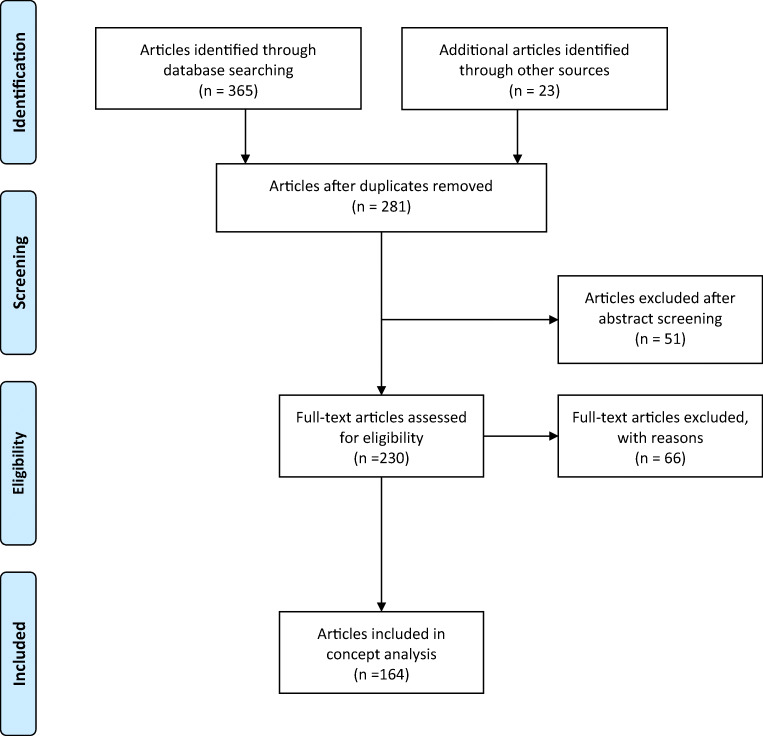
Data search and selection process

A three-strand approach was used to create a systematic search. An initial scoping search was run in PubMed and CINAHL to identify appropriate control language using MeSH and CINAHL headings. Control language is the language of the topic established at the start of a systematic search, against which other terms which emerge are mapped against. This includes developing a list of synonyms and consideration of reference and non-reference words for the search. A secondary scoping search was then conducted identifying appropriate keywords related to the following: technology dependence, technology dependent, complex care needs, complex medical care needs, complex healthcare needs, children with special healthcare needs, medically fragile, and medically complex children. The final search was run in PubMed, CINAHL, and PsycINFO using a combination of the keywords and control language. The search was limited to English-language literature published over the last 30 years up to the 31st of December 2019. The reference lists of the resulting articles were reviewed to identify any other pertinent articles. An additional hand search was conducted and a grey literature search was completed using OpenGrey, the Systems for Information on Grey literature in Europe (SIGLE), World Health Organization (WHO), National Technical Information Service USA (NTIS), and the National Academies Press. (Fig. 1) Duplicate articles were removed from the search and the remaining abstracts and full texts were reviewed to ensure they included reference to technology dependence. Those that did not meet these criteria were not included in the concept analysis.

### Data extraction and analysis

**Fig. 2:**
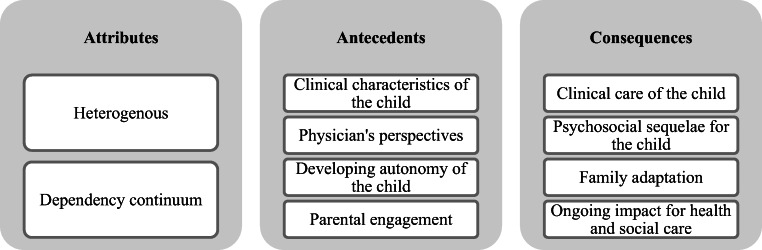
Key characteristics of the attributes, antecedents, and consequences of technology dependence required to sustain a child’s life

The majority of the 164 articles identified were from North America and Europe and the majority of papers are from the last 10 years, reflecting the growing population of children requiring technology to sustain their lives. Each of the articles was analysed for data relevant to the (1) attributes, (2) antecedents, and (3) consequences of a child’s dependence on medical technology to sustain life using a coding framework based on Rodgers’ evolutionary method (Table 2). To guide the data analysis process, a set of specific questions were formulated for each category of data set out by Rodgers (Table 3) [9]. It was important to develop a framework specific to the topic of this concept analysis to ensure a clear focus on the specific areas of interest in the review of the literature. The framework was reviewed by MBr and DA, who then organised recurring themes into each category (attributes, antecedents, and consequences) (Fig. 2). In this way, the structure of the findings below is based on these three categories set out by Rodgers [9].

**Table 2:** Coding framework based on Rodgers’ evolutionary method

Number	Question
1.	What are the key attributes of a child’s dependence on medical technology?
2.	Which factors (antecedents) are proposed to precede technology dependence?
3.	What are the consequences of a child’s dependence on medical technology?

**Table 3:** Guiding questions used during the data analysis phase

Category	Guiding question
Surrogate terms	What other words say the same thing? Is this word/term referring to technology dependence?
Related concepts	Does this term bear any relationship to technology dependence?
Attributes	What are the characteristics of technology dependence, as outlined in this paper? What is the author discussing/describing?
Antecedents	What is happening when technology dependence is initiated? What happens before technology dependence is initiated?
Consequences	What happens after technology dependence is initiated? What happens as a result of technology dependence?

## Findings

### Key attributes of technology dependence required to sustain a child’s life

Synthesis of themes from the literature identified key attributes of technology dependence in children to sustain their lives as heterogenous with a dependency continuum.

#### Heterogenous

Clinical technological dependence in children spanned a wide range of support for multiple human systems. They included oxygen support, invasive and non-invasive mechanical ventilation, pacemaker, implantable cardioverter defibrillators (ICD), ventricular drains, intravenous drugs, intravenous nutrition, gastrostomy or jejunostomy, ileostomy, colostomy, urethral catheterisation, and dialysis [7, 11–17]. We found that the majority of papers referred to children who were technology dependent as children who predominantly required respiratory or cardiac support in addition to requiring additional technological supports to support their wider clinical care needs including nutrition and gastrointestinal function.

#### Dependency continuum

The length of time a child may be dependent on clinical technology to sustain their life can vary, depending on the range and severity of their illness. Children may be dependent on a single device or multiple devices for a defined period, or they may be dependent on clinical technology for a longer period of time, depending on the complexity of their clinical presentation [12, 18]. Some children have the potential of being increasingly technology dependent as their illness progresses, depending on their comorbidities [19–25]. This often emerged in the literature related to specific care transitions including from hospital to home [26–33] and moving from children’s services to adult services [32, 34–37].

### Antecedents of technology dependence required to sustain a child’s life

The conceptual analysis identified four domains regarding antecedents of technology dependence: clinical characteristics of the child, physician perspectives, the developing autonomy of the child, and parental engagement.

#### Clinical characteristics of the child

Specific foreground clinical characteristics which may lead to the initiation of technology dependence are identified in the literature. This includes genetic disorders, congenital disorders, issues related to prematurity or perinatal trauma, acquired external causes such as neoplasia, near drowning or trauma, following prolonged resuscitation, perisurgical anoxia, accidental suffocation, renal failure, and degenerative neurological conditions [11, 13, 24, 38–40].

#### Physician perspectives

The impact of different physician perspectives on care delivery is evident in the literature [38]. Some suggestions for varieties in perspectives include prognostic uncertainty and varying perspectives on the meaning of death of a child in their care [41, 42]. The estimation of prognosis is a significant factor when technology dependence is initiated [43–46]. Where there is prognostic uncertainty, this may be compounded by consideration of the potential opportunities that may emerge with future medical technological advances [17]. On the other hand, physicians who are over pessimistic in their prognostication may not offer hope for survival [47]. It has been suggested that varying perspectives on the initiation of technology dependence may be related to physicians’ views of death; for example, one study review showed that 68% of physicians regarded their patients’ deaths as a personal failure [48].

#### Developing autonomy of the child

Care delivery to children is unique in that those receiving care are developmentally dynamic and the autonomy of those receiving care must be considered [49–57]. A predominant paternalistic stance is evident in that much of the literature in this area focuses on the perspectives of the clinical team or the parents and there is evidence that children are often excluded from the decision-making process [58, 59]. This may be explained by a priori beliefs of the value of a child’s opinion or on limited belief of the importance of the chronological age or developing abilities of children as they age [60, 61]. Literature that espouses increased autonomy of the child in decision-making argues that it can increase the child’s trust and enhance the child-physician relationship [54, 55, 57, 62–66].

#### Parental engagement

The concept analysis found multiple concurrent issues that affect parents when technology dependence is initiated for their child. Parents may be influenced by previous experiences, for example if they have another child with a similar condition, how they experienced the care of that child and whether that child is still alive or has died [39]. They may also be so consumed by their immediate concerns for the child’s survival that they may not fully understand the options available for their child’s care [39, 67, 68]. The literature is replete with stressors of the parents at such critical junctures in care delivery including a feeling of lack of control over the ongoing instability of the child, insomnia, poor diet, and exhaustion [69].

A number of potential organisational challenges were also identified, including the potential for miscommunication of information to parents when more than one clinical team is involved [70, 71]; influences of organisational culture on the degree of choice parents can exercise and the power dynamic between the parent/child and physician in decision making [72]; and coercion or pressure put on parents to make quick decisions when decisions are time-sensitive, which can limit the level of communication and engagement to support parents [39]. Supportive measures identified for parents during this time include having a dedicated coordinator to manage the various care communications; this could facilitate more thorough communication of decisions around care delivery and lead to more informed conversations engendering greater trust with families [17, 73].

### Consequences of technology dependence required to sustain a child’s life

Finally, the consequences of the concept were identified. These are the factors (consequences) that provide biopsychosocial context *beyond* the initiation of technology dependence to sustain a child’s life and the phenomena that occur consequently. The conceptual analysis identified four domains of consequence of the initiation of technology dependence: clinical care of the child; psychosocial sequelae for the child; family adaptation; and ongoing impact for health and social care.

#### Clinical care of the child

A literature review identified multiple issues specific to the clinical care of a child who is dependent on technology to sustain their life. They can have frequent clinic visits, are frequently hospitalised, and have a high risk of critical illness [5–7, 74–76]. These children also have more visits to the Emergency Department than a well child [14, 75, 77–79]. This rate of attendance is often higher than the rates of attendance of elderly patients over 85 years of age [75]. Higher rates of visits are associated with greater distance from the hospital and being a younger child and having a large number of medications [24, 80–82]. Children who are technology dependent may have longer stays in PICU, they are more likely to be readmitted to a PICU during a hospital stay, new morbidities often emerge following admission and readmission to PICU, and they are more likely to die after a prolonged stay in PICU than a child with an acute illness [5, 6, 83–85]. Other clinical issues can include device-related complications; for example, for a child who has an ICD, this may include lead dysfunction, risk of infection, and/or battery depletion [86].

#### Psychosocial sequelae for the child

There was limited attention paid in the literature to the psychological sequelae for a child who is technology dependent. Protective factors against negative psychological sequelae for children assisted by technology include higher cognitive functioning of the child and greater social functioning of the parents [87, 88]. Some-illness specific issues were found. For example, where low health-related quality of life (HRQoL) was found for children who were technology dependent, the lowest scores were found for children who were technology dependent and also had a neurological impairment [88]. In addition, children who had ICDs were found to have a high potential for anxiety, depression, and post-traumatic stress disorder [16, 89, 90] and overall children with pacemakers were found to have lower HRQoL scores than other children with chronic cardiac disease who were not dependent on a rhythm device [91]. It was also found that females and non-Caucasian children living with an ICD had higher prevalence of anxiety and depressive disorders than other children living with the same device [89, 92].

#### Family adaptation

The adaptation of a child’s family to their technology dependence is well documented for children living assisted with respiratory support, though much less so for other technology. The stress of moving out of PICU and the realignment of care expectations has been identified as a period of significant stress for parents [71]. General concerns when planning to move to home includes parents’ stress about becoming a clinician in the home and stress about the potential for equipment malfunction [24, 68, 71, 93–107]. Specific challenges identified include grieving for a well child [71]; learning to master care delivery in a variety of settings [108]; guilt over having less time with the other children at home or relying on them for assistance in care giving [109]; causing pain to their child when carrying out clinical procedures [109–112]; concern over sleep disturbance [108, 113]; and difficulty accessing and delivering a large number of medications [24].

A number of papers highlight how parents obtain a sense of control as they adapt their role as primary care givers of a child who is technology dependent. This includes focusing on becoming an expert carer, focusing on the child’s achievements, and the importance of their spirituality or religion to help them cope [102, 114]. Resilience training has been found to have a positive effect on parents’ ability to cope [115]. However, while parents often become very good clinical problem solvers [112], they have identified specific areas for greater support when planning for discharge. This includes support for ongoing self-directed learning once they are at home, greater support to deal with the myriad of financial concerns, and greater flexibility in the level and amount of care delivery made available to them [71, 101, 105–107, 116]. The impact on siblings adapting to living with a child who is technology dependent is increasingly being documented in the literature. This includes a focus on the positive aspects of adaption such as the development of a strong attachment and having protective tendencies towards their sibling [117]. Negative aspects have also been identified, including a risk of isolation, missing out on social and family events, and risk of psychological distress as the family adapts to a new way of being [117–119]. Earlier papers on this topic urged caution over placing extraordinary burdens on parents and families by the introduction of life-sustaining technology in the home [120]. More recently, there is a very clear impetus internationally to encourage care of the child as close to home as possible and preferably in the home [121, 122].

#### Ongoing impact for health and social care

The variety of challenges for health and social care delivery, related to the increasing number of children who require technology to sustain their lives, are well documented in the literature. This includes challenges in the development of integrated care for this cohort of children and co-creation of integrated care with children and their families [106, 107, 113, 123]. Care delivery across acute and community care services can be complicated by inconsistent standards for discharge to home [107, 124–127]. Specific areas that could enhance care delivery include enhanced access to specialist care, including same-day appointments to appropriately trained physicians in the community [80, 128, 129]; increased use of telehealth, electronic records, and patient summaries [130, 131]; specialist home care visits in the initial week following discharge to home [81]; and increased access to respite care in and out of the home [103, 113, 130–136]. Access to, and governance of, appropriately trained nurses is identified as an ongoing challenge as this population of children grows [5, 35]. Specifically, there is an increasing call for advanced practice nurses across various healthcare sectors to care for children who are technology dependent [88, 103, 135–137] and the need for an increased number of school nurses to support access to education for these children [138–141]. Children dependent on technology are living longer and this impacts on their transition to adult services and onward care in adult healthcare; there is a need for enhanced models of transition to adult services with established and standardised protocols [106, 125].

## Discussion

This concept analysis shows that the term technology dependent continues to be widely used as an umbrella term for a large group of children, without much evidence of any critical consideration of its use. The most commonly referenced definition is now 33 years old [8]. We found that literature on technology dependence to sustain a child’s life refers to *a wide range of clinical technology to support biological functioning across a dependency continuum, for a range of clinical conditions. This assistance is initiated within a complex biopsychosocial context and has wide ranging sequelae for the child and family and health and social care delivery.* This highlighted the heterogenous nature of technology dependence, the fact that children could be assisted by one or more devices and the fact that the length of time for this assistance can vary*.* We also found that the language around technology dependence in the literature to date is very problem focused. The majority of work focuses on the negative sequelae of using technology to assist a child. This includes challenges with decision-making, organisational culture, frequency of hospitalisation, psychological challenges and concerns around family adaptation, and ongoing access to health and social care [6, 59, 90, 96, 113].

Twenty years ago, Nelson [142], in a chapter titled ‘The Ventilator/Baby as Cyborg: a Case Study in Technology and Medical Ethics’, suggested that technology dependence would eventually be viewed as routine care. The question of how we understand this coexistence of human beings with the opportunities posed by advancing technological augmentations is a significant issue [143–145]. An important step is critically reviewing the use of the term technology dependent. There is a need for more contemporary language that is more solution focused and child-centric. Our findings point to the redundancy of the term technology dependent, a mechanical term that continues to be used to group together a growing population of children who are assisted by technology in a myriad of ways, who adapt to, and function with, this assistance very differently. This is essential for research that is required to illuminate coping strategies and adaptation of children and their families to assistance from technology. The use of such a mechanical phrase can also detract from seeking to understand more about the phenomena happening when the use of technology is initiated, the absence of which can lead to the potential for more anecdote and personal opinion to influence actions, than empirical evidence. This is the focus of TechChild, a programme of research funded by the European Research Council which asks *Just because we can, should we? An anthropological perspective on the initiation of technology dependence to sustain a child’s life.* The overarching aim of this project is to specifically explore influences on the initiation of technological assistance and to develop a theory to explain the initiation of this technology in the context of contrasting health, legal, and socio-political systems. Within this 5-year programme, the terminology in use will be examined further and the current paper provides a foundation to this work.

## Limitations

No specific international guidelines emerged from our search of the literature on the use of the term technology dependent. This is likely to be due to the fact that clinical guidelines predominantly focus on specific clinical presentations instead of using more broad terminology. We found that the term technology dependent encompasses a broad span of clinical areas and specialties, though the majority of papers referred to children who require predominantly respiratory or cardiac support. It is possible that some specific characteristics of other groups of children assisted by technology were not identified in this concept analysis, though the final definition may still be pertinent to the wider group of children who are assisted by technology.

## Conclusion

In an era where interventionist medicine is increasingly available for ever more medically fragile children, this concept analysis is timely. We found that the term technology dependent refers to *a wide range of clinical technology to support biological functioning across a dependency continuum, for a range of clinical conditions. They are initiated within a complex biopsychosocial context and have wide ranging sequelae for the child and family and health and social care delivery*. The concept analysis highlighted that this term is predominantly portrayed in the literature as a very problem-focused issue. We suggest that the term is increasingly redundant and objectifies a heterogenous group of children who are assisted by a myriad of technology and who adapt to, and function with, this assistance in numerous ways.

## Abbreviations

ICD: Implantable cardioverter defibrillators
HRQoL: Health-related quality of life
PICU: Paediatric intensive care unit
